# Bacteriophages Targeting an Emerging *Pantoea* spp. for Rice Disease Biocontrol

**DOI:** 10.3390/v18101061

**Published:** 2026-09-24

**Authors:** Patcharin Siringan, Panukar Malawan, Kanhatey Kong

**Affiliations:** School of Food Technology, Institute of Agricultural Technology, Suranaree University of Technology, Nakhon Ratchasima 30000, Thailand; panukar9malawan@gmail.com (P.M.); kongkanhatey@gmail.com (K.K.)

**Keywords:** bacteriophage, *Pantoea* spp., biocontrol, rice disease, siphovirus, myovirus, sustainable agriculture

## Abstract

Bacterial plant diseases caused by the genus *Pantoea* have emerged as severe threats to global rice cultivation, causing destructive conditions such as bacterial leaf blight and grain rot. Due to increasing antibiotic resistance and the environmental hazards of chemical pesticides, bacteriophage-based biocontrol offers a promising, eco-friendly alternative. In this study, we isolated and characterized novel bacteriophages from infected rice paddies in Thailand, using a newly identified host, *Pantoea* sp. isolate 2 based on *16S rRNA* gene sequencing. Transmission electron microscopy revealed that the phages belong to the class *Caudoviricetes*: phage vB_PS-10 displays a siphovirus-like morphotype, whereas phage vB_LX1 exhibits a myovirus-like morphotype. One-step growth analysis of vB_PS-10 uncovered a biphasic propagation pattern with a latent period of 30 min and a burst size of 10 PFU/cell. In vitro lytic assays demonstrated that vB_PS-10 significantly suppressed bacterial growth, achieving a 4 log CFU/mL reduction in host cells after 12 h. Stability assays revealed that vB_PS-10 is highly stable across a broad pH range (6.0–10.0) and resilient at tropical field temperatures (35–45 °C), though sensitive to UV-C radiation and direct sunlight. Finally, a detached leaf assay validated its in planta biocontrol efficacy, showing that phage vB_PS-10 exhibited a significant reduction in disease severity, restricting lesion development to just 13.8% ± 4.37%, whereas the pathogen-only control group showed near-total lesion coverage after 7 days. These findings demonstrate that phage vB_PS-10 has robust potential as a sustainable biological control agent to manage emerging *Pantoea* diseases in rice production.

## 1. Introduction

Rice (*Oryza sativa* L.) is a primary dietary staple for more than half of the global population, making stable rice production critical for securing global food security [1]. However, rice cultivation faces constant threats from various phytopathogens that cause severe crop losses worldwide [2]. Recently, bacteria belonging to the genus *Pantoea* have emerged as a significant threat to rice health [3]. *Pantoea* species, such as *Pantoea ananatis*, are responsible for highly destructive conditions including bacterial grain rot, leaf blight, streaks, spots, stem and fruit rot, top node necrosis, and grain discoloration, which severely reduce both grain yield and milling quality globally [4].

Managing *Pantoea* infections in rice paddies presents a major challenge for modern agriculture. Current management relies heavily on chemical pesticides and antibiotics; however, heavy chemical applications lead to rapid resistance development in bacterial populations [5]. Furthermore, chemical residues pose severe risks to environmental health, disrupt non-target ecosystems, and contradict sustainable agricultural practices [6]. Consequently, there is an urgent need for eco-friendly, highly specific alternative strategies to control emerging bacterial diseases in rice [7].

Bacteriophages (phages) offer a promising solution as targeted biological control agents [8]. Phages are bacterial viruses that specifically target and kill host bacteria without harming surrounding beneficial microbiomes or eukaryotic hosts [9]. Phage biocontrol offers distinct advantages over traditional chemicals, including the ability to replicate at the site of infection—providing a self-sustaining treatment—and a high specificity that preserves beneficial microbial communities in the rice phyllosphere and rhizosphere [10]. While phage therapy has shown significant success against other prominent rice pathogens like *Xanthomonas oryzae* [11], research on phages specifically targeting rice-pathogenic *Pantoea* species remains limited [12]. Successful biocontrol requires the isolation of novel phages with strong lytic activity and robust stability under field environmental conditions [13].

This study describes the isolation and characterization of a novel bacteriophage targeting pathogenic *Pantoea* spp. isolated from infected rice fields. We evaluated its morphological and physiological characteristics, including thermal and pH stability. Furthermore, we assessed its lytic efficacy against *Pantoea* strains in vitro and in planta. Our findings demonstrate the potential of this phage as an effective, sustainable biocontrol agent to mitigate *Pantoea* diseases in rice production.

## 2. Materials and Methods

### 2.1. Preparation of Bacterial Strains for Bacteriophage Isolation and Propagation

The bacterial strains *Pantoea* spp. (isolates 1 and 2), kindly provided by Asst. Prof. Dr. Komrai Buensanteai, were cultured on 1.5% nutrient agar (NA) using the cross-streak plate method and incubated at 28 °C for 48 h. Single colonies of the bacterial isolates were then inoculated into nutrient broth (NB) and incubated in a shaker incubator at 120 rpm and 28 °C until reaching the mid-log phase OD 600 nm = 0.6–0.7. For long-term preservation, bacterial colonies were inoculated into NB supplemented with 40% glycerol and stored at −20 °C.

### 2.2. Molecular Identification of Host Bacterial Strains

To confirm the identity of the host bacterial strains (isolates 1 and 2), genomic DNA was extracted from overnight bacterial cultures using PureLink™ Genomic DNA Mini Kit 50 Preps (Thermo Fisher Scientific, Inc., Waltham, MA, USA) according to the manufacturer’s instructions. The *16S rRNA* gene was amplified via polymerase chain reaction (PCR) using the universal bacterial primers 27F (5′-AGAGTTTGATCCTGGCTCAG-3′) and 907R (5′-CCGTCAATTCMTTTRAGTTT-3′) [14,15]. PCR amplification of the *16S rRNA* gene was performed in a total reaction volume of 50 µL using a Thermo Scientific Taq DNA Polymerase kit according to the manufacturer’s instructions. Each reaction mixture contained: 5 µL of 10× Taq Buffer (with MgCl_2_); 1 µL of dNTP Mix (10 mM each); 1 µL of forward primer 27F (10 µM); 1 µL of reverse primer 907R (10 µM); 0.25 µL of Taq DNA Polymerase (5 U/µL); 2 µL of genomic DNA template; and 39.75 µL of nuclease-free water. The amplification was carried out in a thermal cycler (Bio-Rad, Hercules, CA, USA) with the following program: an initial denaturation at 95 °C for 3 min; followed by 35 cycles of denaturation at 95 °C for 30 s, annealing at 55 °C for 30 s, and extension at 72 °C for 1 min; with a final extension step at 72 °C for 5 min. The resulting PCR products were stored at 4 °C prior to downstream 0.8% agarose gel electrophoresis. and subjected to Sanger sequencing. The resulting nucleotide sequences were analyzed using the Basic Local Alignment Search Tool (BLASTn) against the National Center for Biotechnology Information (NCBI) database to definitively identify the bacterial host isolates at the genus and species levels.

### 2.3. Bacteriophage Isolation and Propagation

Bacteriophages targeting *Pantoea* spp. were isolated following the method described by Dong et al. [11] with slight modifications. Soil, water, dried rice stubble, dried roots, and dried rice leaf samples were collected from paddy fields in Mueang District, Surin Province (10 soil samples), and Phimai District, Nakhon Ratchasima Province, Thailand. For each sample, a 10 g aliquot (or equivalent volume for water) was suspended in 50 mL of sterile deionized (DI) water and agitated in a rotary shaker at 180 rpm for 3 h. Subsequently, the suspension was inoculated with *Pantoea* spp. isolate 2 and incubated under continuous shaking for an additional 24 h to enrich the phage population. The mixture was then centrifuged at 4500× *g* for 15 min at 4 °C to pellet the soil debris and bacterial cells. The supernatant was collected and filtered through a 0.22 μm sterile syringe filter to obtain a cell-free phage lysate. The presence of bacteriophages in the filtrate was confirmed using the double-layer agar overlay plaque assay [16]. Briefly, a 100 μL aliquot of the filtered lysate was mixed with 500 μL of the *Pantoea* spp. host bacterial suspension in a microcentrifuge tube. The mixture was incubated at 28 °C for 20 min with shaking at 180 rpm to allow phage adsorption. After incubation, the mixture was transferred into 5 mL of molten semi-solid nutrient agar (0.8% agar, maintained at 50 °C), gently homogenized using a vortex mixer, and immediately poured onto a bottom layer of solid nutrient agar (1.5% agar). After the top agar solidified, the plates were incubated at 28 °C for 48 h. The plates were examined for the formation of distinct clear zones (plaques). All assays were performed in triplicate.

Phage purification was performed with modifications to standard polyethylene glycol (PEG) precipitation and cesium chloride (CsCl) density gradient centrifugation protocols [17]. The crude phage lysate 10^8^ PFU/mL was mixed with sodium chloride (NaCl) to a final concentration of 0.5 M, incubated at 4 °C for 60 min, and then centrifuged at 10,000× *g* for 10 min at 4 °C. Polyethylene glycol (PEG-8000) was added to the supernatant to a final concentration of 10% (*w*/*v*). The mixture was gently inverted by hand and incubated at 4 °C for 30 min until a film-like layer became visible. The precipitate was harvested by centrifugation at 10,000× *g* for 10 min at 4 °C. After discarding the supernatant, the resulting pellet was resuspended in Saline-Magnesium (SM) buffer (50 mM Tris-HCl, 100 mM NaCl, 10 mM MgSO4, pH 7.5, with 0.01% (*w*/*v*) gelatin at a 1:1 (*v*/*v*) ratio and allowed to dissolve completely at 4 °C for 24 h). Following incubation, the suspension was centrifuged at 10,000× *g* for 10 min at 4 °C to remove remaining debris. High-purity CsCl (99.9%) was added to the phage suspension to achieve a final density of 1.35 g/mL. The mixture was subjected to ultracentrifugation at 55,000× *g* for 4.5 h at 4 °C. The distinct opalescent phage band formed during density gradient separation was carefully collected using a syringe and used for downstream applications and propagation.

### 2.4. Phage Enumeration and Titration

The concentration of the propagated bacteriophages was determined by counting plaque-forming units (PFUs) using a modified spot assay on double-layer agar plates. The purified phage stock was serially diluted (10-fold) in SM buffer without gelatin. Aliquots of 10 µL from each dilution were spotted onto pre-prepared *Pantoea* spp. double-layer agar lawns [14]. The assays were performed in triplicate. The plates were incubated at 28 °C for 48 h, after which the visible plaques were counted, and the phage titer was calculated and expressed as PFU/mL.

### 2.5. Morphological Analysis by Transmission Electron Microscopy (TEM)

The morphological characteristics of the isolated bacteriophage were examined using negative staining according to the methods described by Atterbury et al. [18] and Hooton et al. [19], with minor modifications. A 10 µL aliquot of the purified phage suspension 10^8^ PFU/mL was deposited onto a piece of Parafilm. A carbon-coated 200-mesh copper grid was gently placed on top of the droplet and allowed to sit for 1 min to facilitate phage adsorption. Excess liquid was carefully blotted away using a piece of sterile filter paper. Subsequently, the grid was placed onto a 10 µL droplet of 2% (*w*/*v*) uranyl acetate (EMS, Hatfield, PA, USA) for 5 min to negatively stain the sample. The remaining staining solution was removed using filter paper, and the grid was allowed to air-dry completely at room temperature. The specimen was visualized using a transmission electron microscope (Talos F200X, Thermo Scientific, Waltham, MA, USA) operating at an accelerating voltage of 120 kV.

### 2.6. One-Step Growth Curve Analysis

A one-step growth curve analysis was performed in triplicate to determine the latent period and burst size of the bacteriophage. A 10 mL culture of *Pantoea* spp. was grown in nutrient broth (NB) at 28 °C until it reached the early exponential growth phase. The definitive bacterial cell count was verified on 1.5% nutrient agar (NA) plates, and the suspension was adjusted with fresh NB to a final density of 10^6^ CFU/mL. The purified phage stock was then inoculated into the bacterial culture at a multiplicity of infection (MOI) of 0.01. The mixture was incubated in a shaker incubator at 28 °C for 150 min. Aliquots of 1 mL were sampled at 10 min intervals and transferred to 1.5 mL microcentrifuge tubes, and then 1% (*w*/*v*) chloroform was added as a final concentration for lysis of phage progeny inside the bacterial host. The samples were immediately centrifuged at 10,000× *g* for 10 min at 4 °C to separate unabsorbed phages and host cells. The resulting supernatant containing the free phages was serially diluted in SM buffer (pH 7.5). Phage titers at each time point were determined via the spot assay by depositing 10 µL of each dilution onto pre-prepared *Pantoea* spp. double-layer agar lawns. The plates were incubated at 28 °C for 48 h, after which the plaques were enumerated to calculate the phage titer in PFU/mL.

### 2.7. In Vitro Lytic Efficacy of Phage vB_PS-10 Against Pantoea spp.

The in vitro bacterial growth inhibition efficiency of bacteriophage vB_PS-10 was evaluated against *Pantoea* spp. host strains. The bacterial culture was grown in nutrient broth (NB) in a shaker incubator at 28 °C for 24 h. The optical density of the bacterial suspension was adjusted with fresh NB to an OD 600 nm of 0.3. Aliquots of 1.5 mL of the adjusted bacterial suspension were transferred into three sterile test tubes. Subsequently, 100 µL of the purified phage suspension was inoculated into each tube to achieve a multiplicity of infection (MOI) of 1. A bacterial suspension without phage inoculation served as the negative control. The mixtures were incubated at 28 °C with gentle continuous agitation for 12 h. Bacterial growth kinetics were monitored by measuring the absorbance at OD 600 nm every hour using a spectrophotometer. Simultaneously, the viable bacterial cell count (CFU/mL) and phage titer (PFU/mL) were determined at each time point through serial dilutions and plaque/colony counting assays as described in the previous sections.

### 2.8. Stability of Bacteriophage vB_PS-10 Under Various Environmental Conditions

#### 2.8.1. Thermal and pH Stability

The stability of phage vB_PS-10 was evaluated under different temperature and pH regimes. For the thermal stability assay, a purified phage suspension with an initial concentration of 10^9^ PFU/mL was incubated at various temperatures (4, 35, 45, and 65 °C); the assay was performed in triplicate. Aliquots were sampled at designated time points (0, 15, 30, and 60 min) to determine the residual phage titer. For the pH stability assay, a 100 µL aliquot of the stock phage suspension 10^9^ PFU/mL was inoculated into 900 µL of SM buffer pre-adjusted to distinct pH levels ranging from 2 to 12 (using NaOH or HCl solutions); this was also performed in triplicate. The mixtures were incubated at room temperature, and samples were withdrawn at 1 h and 18 h post-incubation to determine surviving phage counts.

#### 2.8.2. Ultraviolet (UV) Radiation and Direct Sunlight Stability

To evaluate UV radiation tolerance, a 254 nm UV light source inside a laminar flow hood was utilized. Aliquots of 1 mL of the phage suspension 10^6^ PFU/mL were poured into three separate Petri dishes and exposed to the UV light from a fixed distance of 90 cm. Samples were harvested after 2, 5, and 10 min of continuous exposure to evaluate viability. For direct sunlight stability, a 1 mL aliquot of the phage suspension 10^8^ PFU/mL was placed in three covered Petri dishes and exposed to natural outdoor sunlight. The experiment was conducted during peak daylight hours from 12:30 PM to 3:30 PM. Phage samples were collected at 1 h intervals throughout the exposure period to monitor the degradation rate. All assays across all environmental variables were performed in triplicate, and residual titers were determined using the double-layer agar overlay method.

### 2.9. In Planta Biocontrol Efficacy of Phage vB_PS-10 Using a Detached Leaf Assay

The capacity of bacteriophage vB_PS-10 to reduce disease severity caused by *Pantoea* spp. infection was evaluated using a detached leaf assay [20]. Healthy leaves were harvested from 20-day-old rice plants and cut into 5 cm segments. The leaf segments were surface-disinfected by washing with 70% (*v*/*v*) ethanol and rinsed thoroughly with sterile distilled water. Bacterial inoculation was performed using a sterile syringe filled with a *Pantoea* spp. suspension adjusted to a concentration of 10^6^ CFU/mL. A 0.1 mL aliquot of the bacterial suspension was infiltrated into three leaf segments were placed on a Petri dish in triplicate. For the phage treatment group, a 0.1 mL aliquot of the purified phage suspension 10^6^ PFU/mL was immediately infiltrated into the exact same inoculation site. The treated leaf segments were placed in sterile Petri dishes containing filter paper moistened with sterile water to maintain high humidity. The plates were incubated at 28 °C for 7 days. At the end of the incubation period, the lesion length was measured using ImageJ (version 1.54g), and the total diseased area on each rice leaf segment was calculated to determine biocontrol efficacy. The lesion area was calculated as follows: lesion area (%) = [lesion area (cm^2^)/total leaf area (cm^2^)] × 100. The statistical analysis was performed using a paired *t*-test to compare the control group and the phage treatment group.

## 3. Results

### 3.1. Taxonomic Verification of the Bacterial Host Isolate

The taxonomic classification of the bacterial strain used as the host for bacteriophage isolation was evaluated via partial *16S rRNA* gene sequencing. Standard nucleotide BLAST (BLASTn version 2.16.0+) analysis revealed that the isolate belongs to the genus *Pantoea*, displaying the highest sequence homology to *Pantoea agglomerans* strain L3 (Accession: PQ459961.1), *Pantoea brenneri* strain PB7_77 (Accession: PP422059.1), and *Pantoea graminicola* strain JY-2 (Accession: PZ311982.1). The sequence alignment exhibited a percent identity ranging from 94.05% to 95.66%, with a query coverage of 74% to 79%. A neighbor-joining phylogenetic tree was constructed to visualize its relationship with other reference *Enterobacteriaceae* (Figure 1). Due to the partial nature of the sequence alignment and the distinct branching pattern at the root of the cluster, the host isolate was conservatively designated as *Pantoea* sp. isolate 2 throughout this study and was successfully utilized for subsequent phage propagation.

### 3.2. Isolation and Purification of Bacteriophages

Screening of the 10 environmental soil samples collected from Mueang District, Surin Province, yielded a single positive sample containing a distinct lytic phage. This initial isolation was confirmed by the observation of a single clear plaque on the host bacterial lawn. Following successful propagation, the uniform plaque morphology of this isolate was documented (Figure 2). To ensure a genetically homogeneous population for downstream characterization, a single well-isolated plaque was picked from each plate. This clonal purification process via the double-layer agar overlay assay was repeated for three consecutive rounds. The pure phage isolates were then propagated to generate high-titer working stocks for subsequent morphological, physiological, and genomic characterization. This isolated phage (Figure 2A) was purified and designated vB_PS-10. Phage vB_PS-10 was propagated to obtain a high titer of approximately 10^9^ PFU/mL. Concurrently, screening of the 12 agricultural samples (comprising soil, water, rice stubble, dried rice leaf, and dried roots); *n* = 3 per sample type collected from Phimai District, Nakhon Ratchasima Province, successfully yielded another phage isolate. The characteristic plaque morphology of this second collection is shown in Figure S1. The plaque morphology of isolate vB_LX1 from dried rice leaves is shown in Figure 2B. The phage titer of vB-LX1 was around 10^7^ PFU/mL. Consequently, phage vB_PS-10 was prioritized for further characterization and in planta application. This decision was based on its superior propagation and efficiency in in vitro lytic assays, alongside the most robust bacterial host reduction among the isolated pool.

### 3.3. Morphological Characterization via Transmission Electron Microscopy (TEM)

Transmission electron microscopy (TEM) examination revealed distinct structural archetypes for the two isolated bacteriophages (Figure 3). Phage vB_PS-10 exhibited an isometric hexagonal head measuring approximately 48.27 ± 1.59 nm in diameter, attached to a long, flexible, non-contractile tail measuring 129.29 ± 6.69 nm in length (Figure 3A). Based on these morphological hallmarks, phage vB_PS-10 displays a characteristic siphovirus-like morphotype, historically classified under the family *Siphoviridae*. In contrast, phage vB_LX1 possessed an isometric polygonal head measuring roughly 44.10 ± 1.54 nm in diameter, connected to a long, straight, rigid, and contractile tail with a length of 105.89 ± 2.49 nm (Figure 3B). These distinct structural traits identify phage vB_LX1 as a myovirus-like morphotype, a group historically belonging to the family *Myoviridae*. Both phages are taxonomic members of the class *Caudoviricetes*, representing double-stranded DNA tailed bacterial viruses.

### 3.4. Growth Kinetics of Phage vB_PS-10

The replication kinetics of phage vB_PS-10 were determined via a one-step growth curve analysis (Figure 4). The phage displayed a latent period of approximately 30 min, followed by an initial burst size calculated to be 10 PFU per infected bacterial cell. Interestingly, the growth profile revealed a biphasic replication pattern characterized by two distinct, consecutive rises in phage titer before entering the plateau phase. The final viral concentration stabilized during the plateau phase at approximately 7.2 log PFU/mL.

### 3.5. In Vitro Lytic Efficiency and Bacterial Growth Suppression

The capacity of bacteriophage vB_PS-10 to suppress the growth of its host bacterium, *Pantoea* sp., was demonstrated through optical density monitoring and viable cell enumeration (Figure 5). When inoculated at a multiplicity of infection (MOI) of 1.0, the phage-treated bacterial cultures exhibited a statistically significant decrease in turbidity (OD 600) compared to the uninfected control group, which showed typical exponential growth. This lytic efficacy was further validated by viable cell counts. Following 12 h of incubation at 28 °C, the host bacterial population in the phage-treated group was reduced by approximately 4 log CFU/mL relative to the non-treated negative control. Moreover, an increased MOI resulted in a rapid reduction in optical density at OD 600 (Figure S2). These concurrent findings from both absorbance profiles and colony-forming unit determinations confirm that phage vB_PS-10 possesses robust and rapid in vitro lytic activity against pathogenic *Pantoea* sp. strains.

### 3.6. Stability Profiles of Phage vB_PS-10 Under Various Environmental Conditions

#### 3.6.1. Effects of pH on Phage Stability

The stability of bacteriophage vB_PS-10 across a wide pH gradient (pH 2–12) was evaluated after incubation at room temperature (Figure 6A). Phage vB_PS-10 exhibited high stability within the neutral to alkaline range of pH 6.0–10.0. In contrast, extreme acidic conditions were highly detrimental, resulting in complete viral inactivation at pH 2.0. However, the phage demonstrated moderate tolerance to mild acidity; survival rates decreased by only 15–18% at pH 4.0–5.0, and by 36% at pH 3.0. At the extreme alkaline end (pH 12.0), the phage titer decreased by 25%. These findings collectively demonstrate that phage vB_PS-10 possesses higher structural stability under alkaline conditions compared with acidic environments.

#### 3.6.2. Thermal Stability Characteristics

Thermal tolerance assays revealed that phage vB_PS-10 maintains robust viability when exposed to temperatures of 4, 35, and 45 °C for up to 1 h (Figure 6B). Exposure to an elevated temperature of 65 °C induced time-dependent viral degradation. Specifically, the phage population declined by 15% during the initial incubation period and continued to drop significantly by 35% after 60 min of continuous exposure. This indicates that while the phage is sensitive to extreme heat, it remains highly stable at 4 °C (the standard storage temperature for experimental preservation) and exhibits excellent resilience at 35 °C and 45 °C. This environmental tolerance is critical, as these temperatures closely mimic the ambient heat and field conditions typical of tropical regions like Thailand.

### 3.7. Stability Profiles of Phage vB_PS-10 Under Ultraviolet (UV) Radiation and Natural Sunlight

#### 3.7.1. Tolerance to Ultraviolet (UV) Radiation

Bacteriophage vB_PS-10 exhibited high sensitivity to ultraviolet radiation (Figure 7A). Because the phage possesses a double-stranded DNA (dsDNA) genome, its genetic material is highly susceptible to structural damage and degradation induced by UV-C light irradiation. Consequently, continuous exposure to 254 nm UV radiation resulted in a progressive decline in viral viability, culminating in a 33% reduction in the active phage titer after 10 min of direct exposure.

#### 3.7.2. Tolerance to Direct Sunlight

The environmental resilience of phage vB_PS-10 was further evaluated under natural outdoor sunlight during peak solar intensity hours (12:30 PM to 3:30 PM), with ambient field temperatures ranging between 45 °C and 47 °C (Figure 7B). The phage population demonstrated a time-dependent decrease when subjected to these conditions. Following 3 h of continuous open-air exposure, the active viral titer dropped significantly by approximately 3 log PFU/mL. This reduction highlights the disruptive impact of combined solar radiation and elevated field temperatures on virion stability over extended periods.

### 3.8. In Planta Biocontrol Efficacy Against Pantoea sp. on Rice Leaves

The potential of bacteriophage vB_PS-10 as a biological control agent to mitigate bacterial leaf blight and grain rot symptoms was validated using an in planta detached leaf assay (Figure 8). Following a 7-day incubation period, rice leaf segments inoculated with *Pantoea* sp. alone exhibited distinct symptoms of bacterial leaf blight and localized necrotic spots. In contrast, leaf segments co-treated with phage vB_PS-10 demonstrated a significant reduction in disease severity, significantly restricting lesion development to just 13.8% ± 4.37% of the leaf area compared with the near-complete coverage (89.87% ± 8.99%) observed in the pathogen-only control group (*p* < 0.05). These findings indicate that phage vB_PS-10 successfully inhibits host bacterial infection within plant tissue, thereby suppressing the development of leaf blight and spotting symptoms. Consequently, these results provide evidence that this bacteriophage could serve as an effective biocontrol agent for managing *Pantoea* infections. However, future studies will focus on simulating the actual ecosystem of a rice field to demonstrate the efficacy of phage vB_PS-10.

## 4. Discussion

The emergence of *Pantoea* species as destructive phytopathogens affecting global rice cultivation demands the development of sustainable, eco-friendly alternatives to traditional chemical treatments. In this study, we successfully isolated and characterized the novel bacteriophage vB_PS-10 and evaluated its therapeutic potential against a pathogenic *Pantoea* strain from Thai rice paddies. Our findings provide valuable insights into the biological attributes of this phage and underscore its capability as a targeted biocontrol agent. A critical finding of this study is the taxonomic status of the host bacterial strain, *Pantoea* sp. isolate 2. Molecular characterization via *16S rRNA* gene sequencing revealed that it shared only 95% sequence identity with *Pantoea agglomerans* (Figure 1). In prokaryotic taxonomy, a *16S rRNA* gene sequence similarity threshold below 98.65% is universally accepted as a strong indicator of a novel species boundary [21]. This indicates that our host isolate represents a previously uncharacterized, novel species within the *Pantoea* genus. The discovery of an emerging rice pathogen with a distinct genetic background mirrors recent reports of diverse, novel *Pantoea* leaf blight outbreaks identified across Asian agricultural zones [22,23,24]. The isolation of phage vB_PS-10 using this unique host highlights the hidden diversity of both phytopathogenic bacteria and their corresponding viral predators in tropical rice ecosystems. Morphological analysis via transmission electron microscopy (TEM) revealed that the two isolated phages, vB_PS-10 and vB_LX1, belong to distinct structural classes within the order *Caudoviricetes*. Phage vB_PS-10 exhibited a classic siphovirus-like morphotype, characterized by an isometric hexagonal head and a long, flexible, non-contractile tail (Figure 3A). Conversely, phage vB_LX1 displayed a myovirus-like morphotype with a rigid, contractile tail (Figure 3B). While myoviruses targeting *Pantoea* have been documented [12,25], siphovirus-like phages infecting this genus remain less common [26,27]. The diversity captured between these two isolates provides excellent candidate strains for future phage cocktail formulations, which typically benefit from combining different viral families to minimize the risk of bacterial resistance development. The replication kinetics of phage vB_PS-10, determined via a one-step growth curve, uncovered a unique biphasic propagation pattern (Figure 4). It demonstrated a short latent period of 30 min and an initial burst size of 10 PFU/cell. The subsequent secondary rise in viral titer before reaching a plateau phase at 7.2 log PFU/mL is characteristic of a double-step growth mechanism. This phenomenon typically occurs in closed in vitro systems when newly released progeny from the first burst cycle immediately infect neighboring uninfected host cells. The in vitro lytic assay demonstrated that phage vB_PS-10 possesses excellent antibacterial efficacy. At an MOI of 1.0, the phage significantly suppressed bacterial turbidity and induced a dramatic 4 log CFU/mL reduction in viable host cells after 12 h of incubation (Figure 5). This profound capacity to decimate host populations in vitro is a fundamental prerequisite for any successful biocontrol agent. For open-field agricultural applications, a phage must survive fluctuating environmental stressors. Phage vB_PS-10 exhibited an outstanding physiological tolerance profile. It remained highly stable across a broad pH range (pH 6.0–10.0), with higher resilience under alkaline conditions than acidic ones (Figure 6A). This alkaline preference aligns well with the physiological realities of certain agricultural soils and cellular environments. Thermally, the phage maintained robust viability at 35 °C and 45 °C (Figure 6B), which is highly significant as these parameters closely mirror the ambient heat and tropical field conditions of Thailand. However, environmental radiation posed a challenge. As a double-stranded DNA virus, vB_PS-10 proved sensitive to UV-C radiation, showing a 33% decline within 10 min (Figure 7A), and a 3 Log reduction after 3 h of direct sunlight exposure (Figure 7B). This vulnerability to solar UV radiation is a well-documented bottleneck in agricultural phage therapy. To translate phage vB_PS-10 into a practical field spray, future optimization must incorporate natural UV-protectant carriers, such as skim milk, sucrose, or biopolymer encapsulation, to shield the viral particles from solar degradation. Finally, the in planta detached leaf assay confirmed the practical utility of phage vB_PS-10. Co-treatment with the phage successfully restricted *Pantoea* sp. infection within the plant tissue, limiting leaf blight lesions and necrotic spots to approximately 13.8% ± 4.37% of the total leaf area over 7 days (Figure 8). This result demonstrates that vB_PS-10 significantly suppresses disease severity and limits symptom progression in rice leaves. Similarly, in planta experiments utilizing phage PanM1EGY treatments in potato tubers, onion bulbs, and garlic cloves showed inhibition of rot disease symptoms caused by *Pantoea ananatis* compared with untreated controls [12]. Azegami and Inoue [28] reported the application of bacteriophage Φ for the biocontrol of the palea browning of rice, caused by *P. ananatis* in the field for three years, resulting in a suppression of disease incidence of approximately 50–86% compared with the control. It was notable that phages can significantly retard bacterial disease in real-world plant systems. However, future studies of vB_PS-10 will utilize both simulated and in-field rice ecosystems to thoroughly evaluate the biocontrol efficacy of this phage under real-world agricultural conditions.

In conclusion, this study demonstrates that the bacteriophage vB_PS-10 could serve as an effective biocontrol agent for managing destructive *Pantoea* diseases in rice cultivation. Characterized by great propagation efficiency, vB_PS-10 consistently achieves a high-titer profile. Furthermore, its biological stability across a wide pH range and under tropical field temperatures highlights its resilience, despite minor reductions in viability when exposed to UV-C radiation and direct sunlight. Moreover, in vitro lytic performance and the in planta detached leaf assay validated the practical utility of vB_PS-10, resulting in reduction in a rice disease. Consequently, future studies will focus on both simulated and in-field rice ecosystems to thoroughly evaluate the therapeutic biocontrol efficacy of this phage under variable, real-world agricultural conditions.

## Figures and Tables

**Figure 1:**
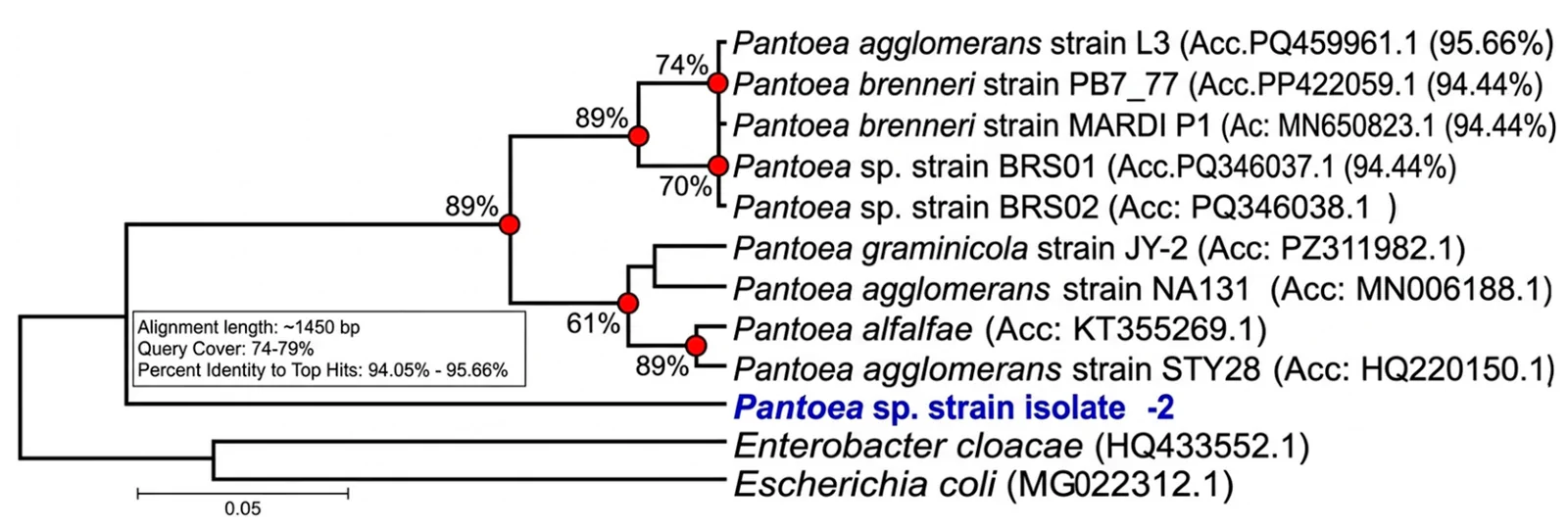
Neighbor-joining phylogenetic tree based on partial *16S rRNA* gene sequences illustrating the taxonomic positioning of *Pantoea* sp. isolate 2 (Query_6324541) relative to closely related, validly described *Pantoea* species. Scale bar represents 0.05 substitutions per nucleotide position.

**Figure 2:**
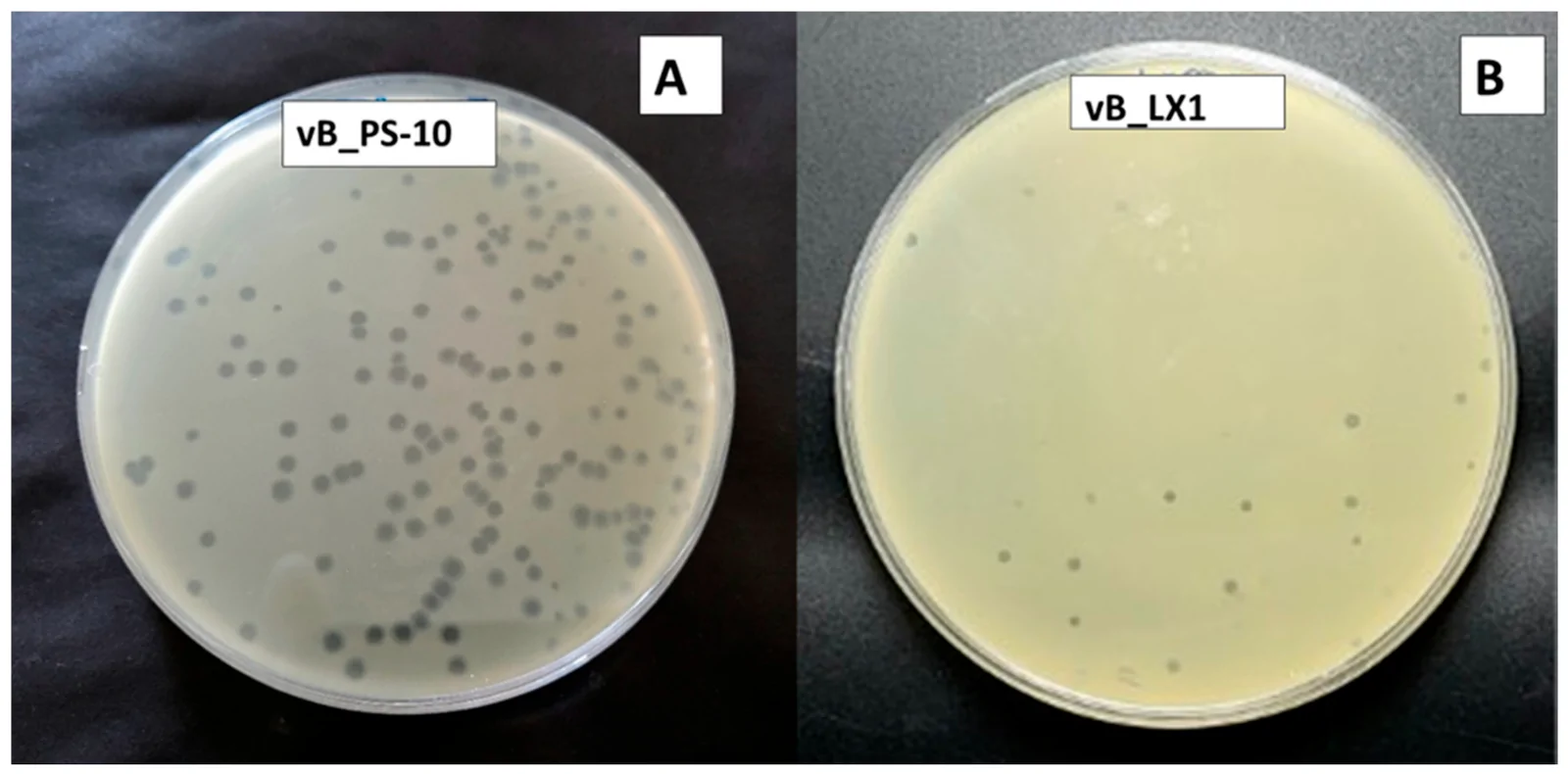
Plaque morphology of the bacteriophage isolated from soil samples in Mueang District, Surin Province, named vB_PS-10 (**A**) and a dried rice leaf sample in Phimai District, Nakhon Ratchasima Province, named vB_LX1 (**B**), visualized on a *Pantoea* sp. isolate 2 bacterial lawn after 48 h of incubation at 28 °C.

**Figure 3:**
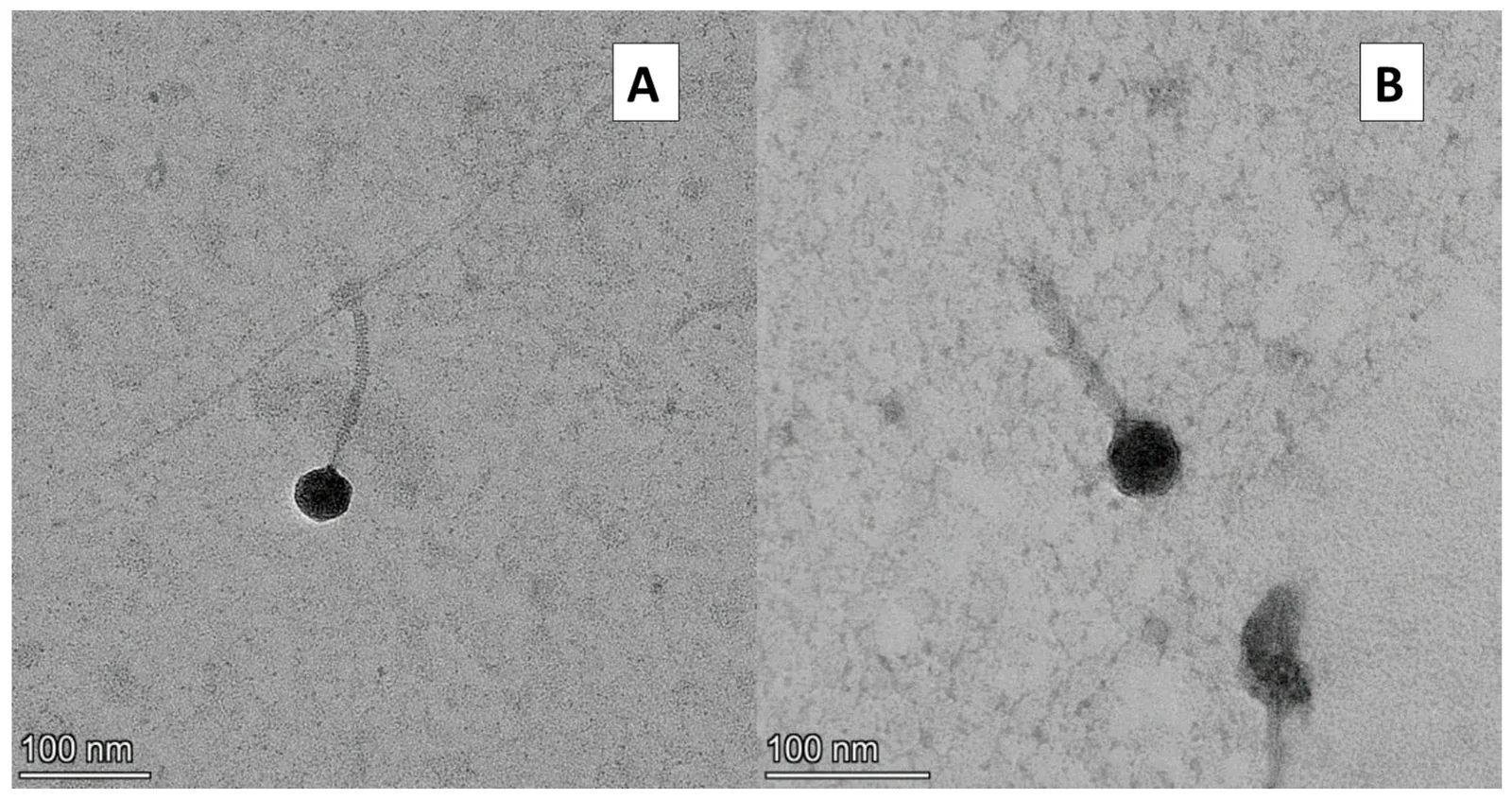
Morphology of purified bacteriophages (**A**) Phage vB_PS-10 (**B**) Phage vB_LX1 observed via transmission electron microscopy.

**Figure 4:**
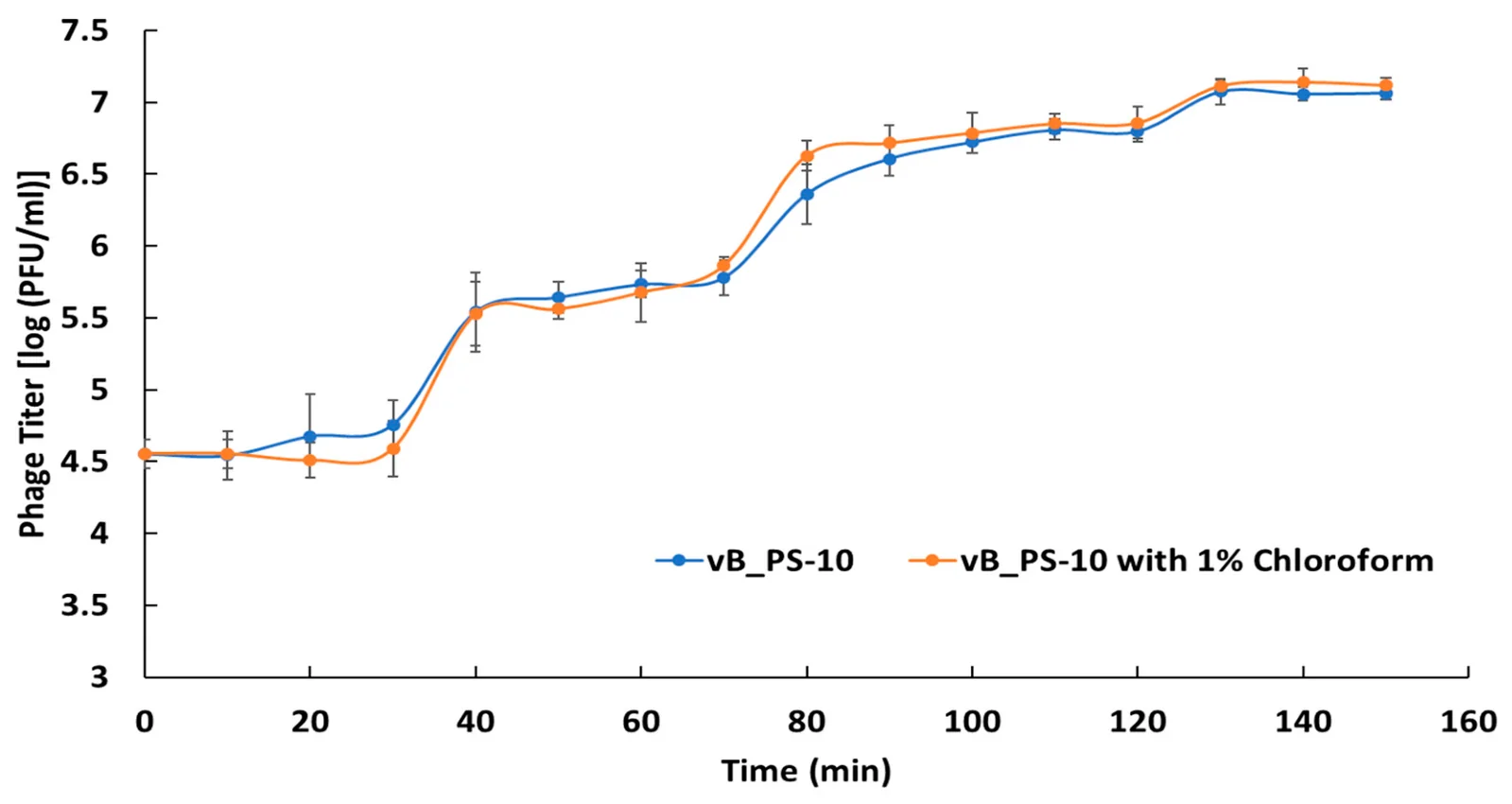
One-step growth curve of bacteriophage vB_PS-10 on *Pantoea* sp. isolate 2 at a multiplicity of infection (MOI) of 0.01.

**Figure 5:**
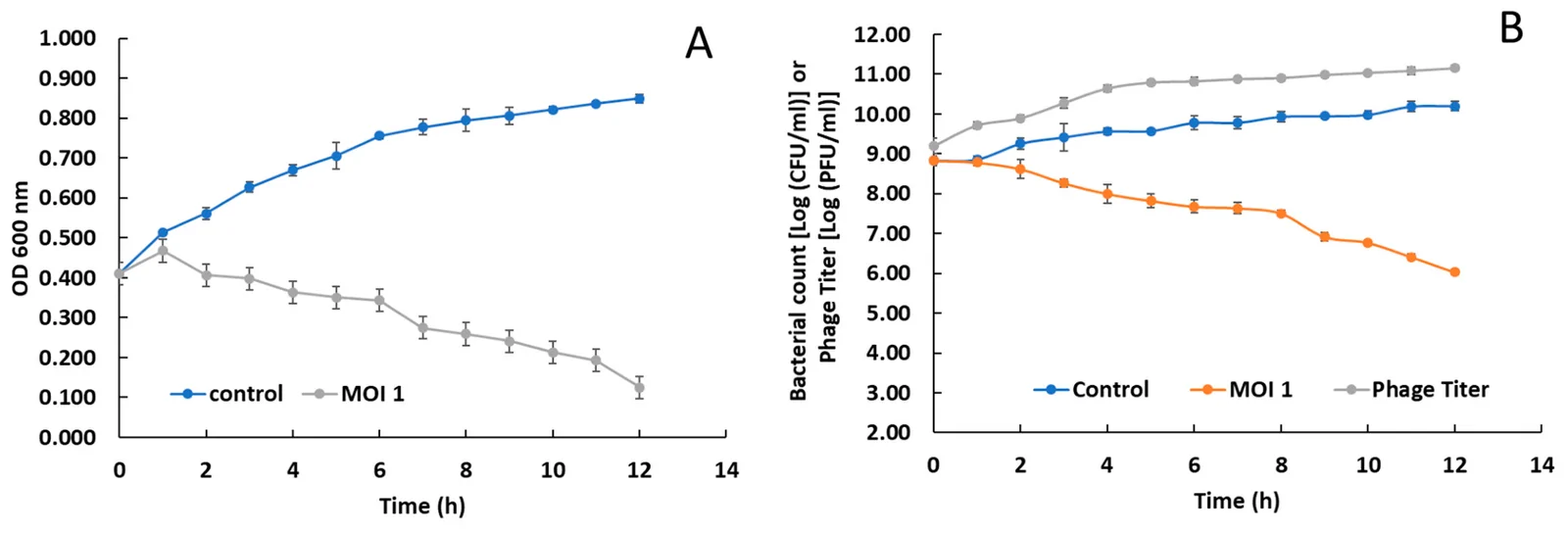
In vitro lysis assay of *Pantoea* sp. isolate 2 treated with bacteriophage vB_PS-10 at a multiplicity of infection (MOI) of 1.085 at 28 °C. Bacterial growth was monitored by measuring the optical density (OD) at 600 nm (**A**) and viable cell count of *Pantoea* sp. isolate 2 compared to the untreated control (**B**).

**Figure 6:**
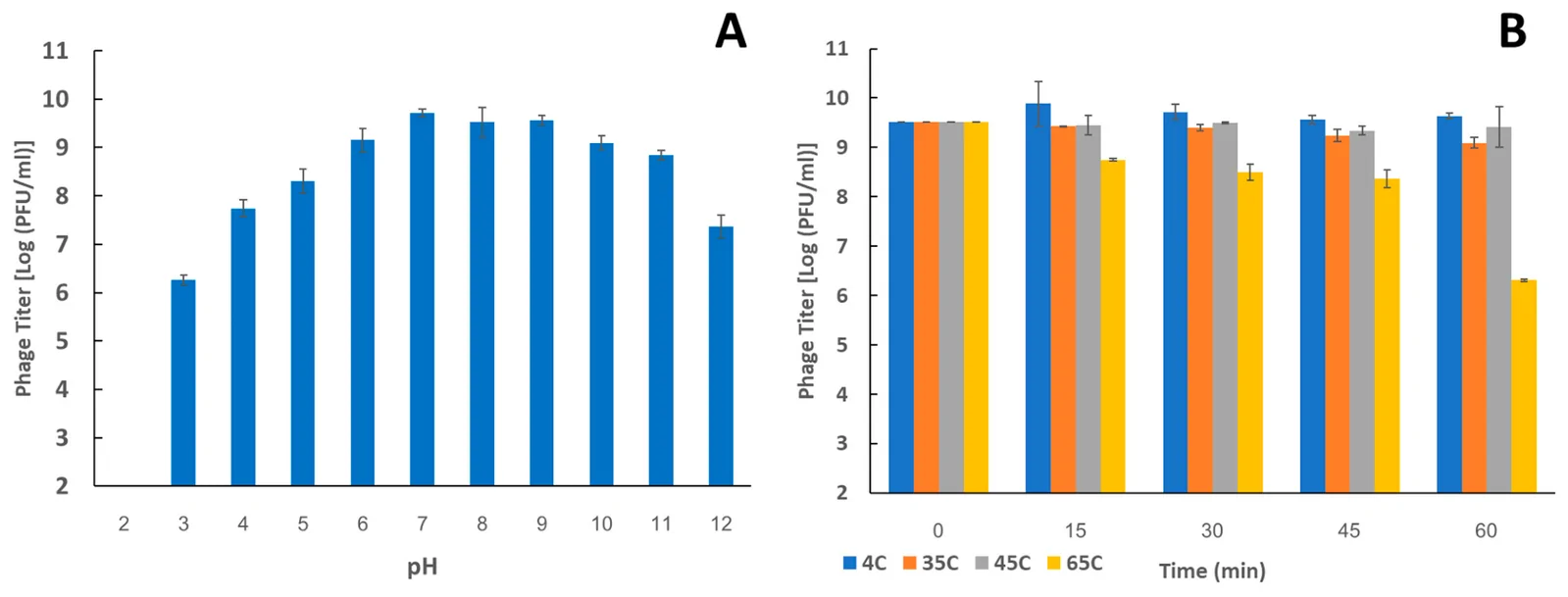
Stability of bacteriophage vB_PS-10 under different pH conditions ranging from 2.0 to 12.0 (**A**) and thermal stability exposed to temperatures of 4, 35, 45, and 65 °C for 15, 30, and 60 min (**B**).

**Figure 7:**
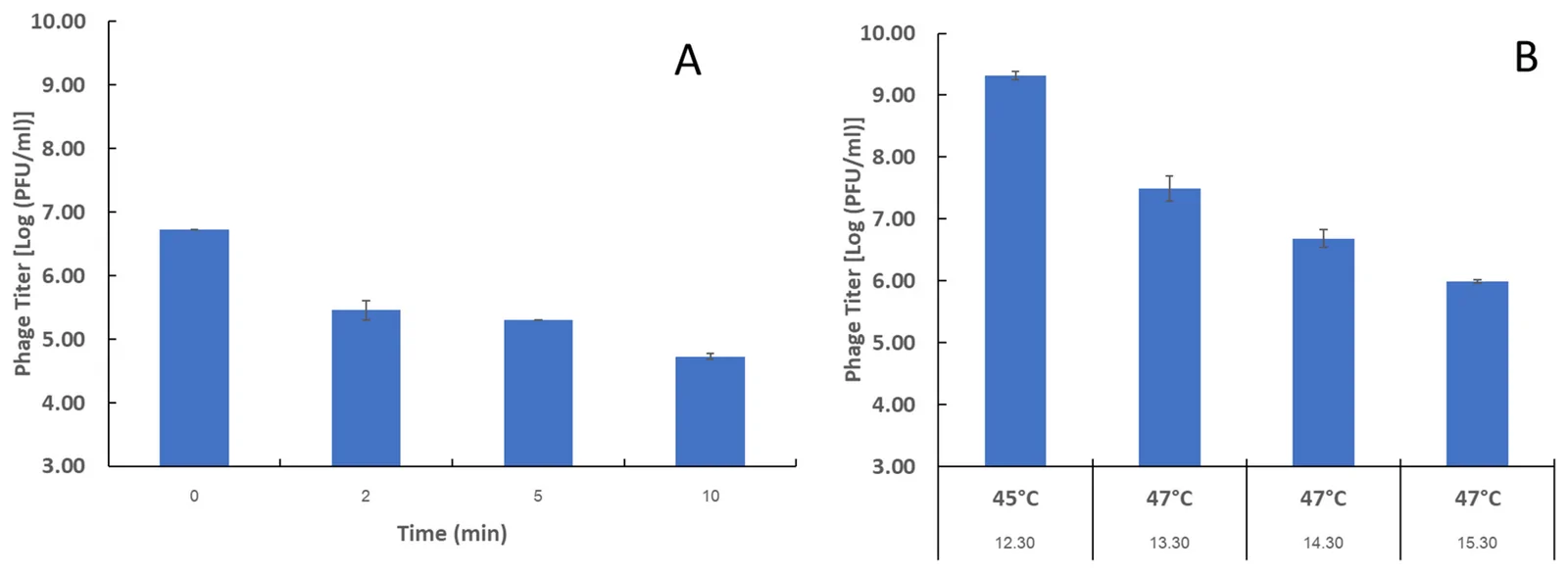
Survival bacteriophage vB_PS-10 under 254 nm ultraviolet (UV-C) radiation (**A**) and under direct natural sunlight exposure from 12:30 PM to 3:30 PM at ambient temperatures of 45–47 °C (**B**).

**Figure 8:**
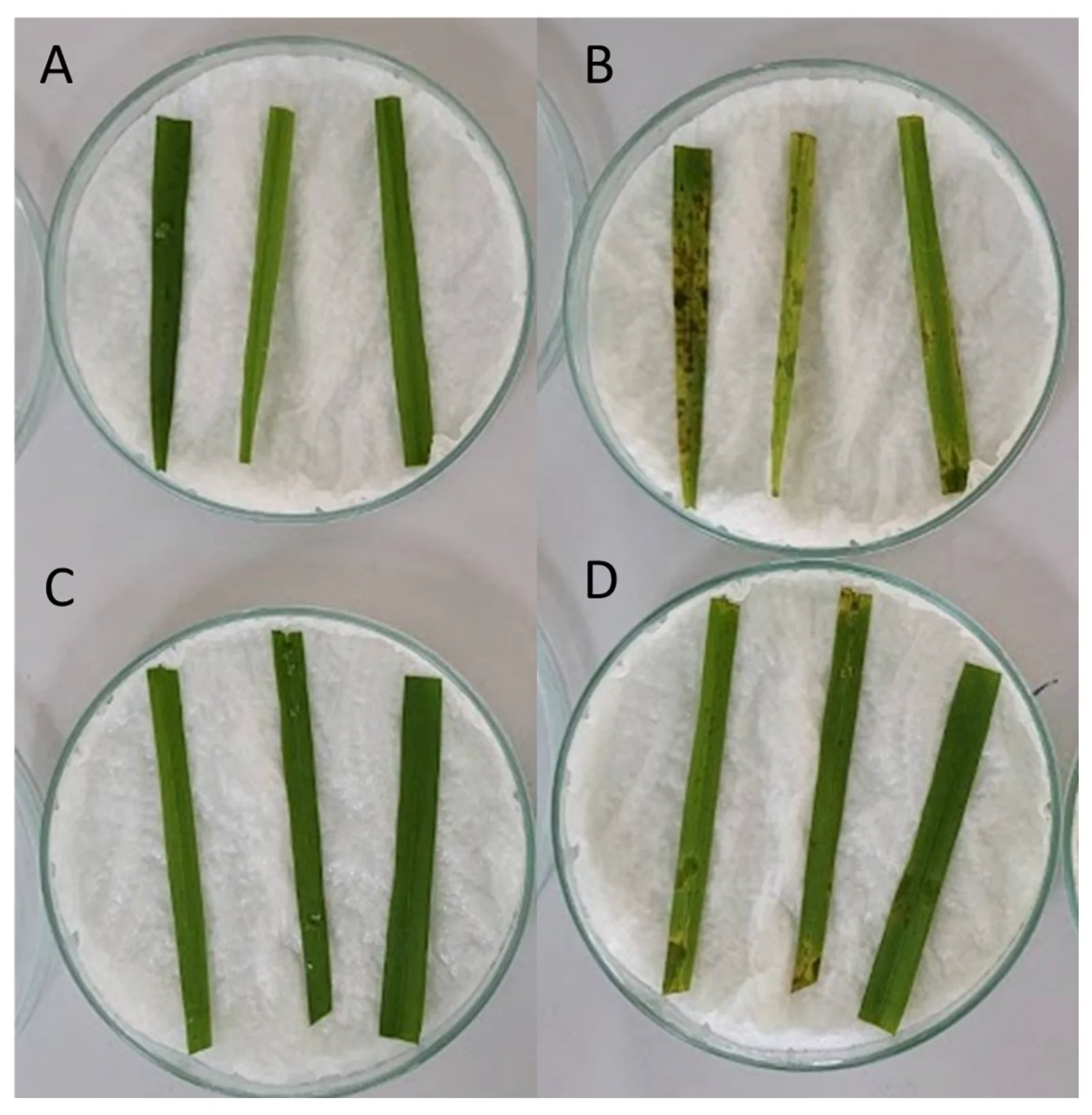
Biocontrol efficacy of bacteriophage vB_PS-10 against *Pantoea* sp. isolate 2 on detached rice leaf segments at the initial point (0 days), and after 7 days of incubation at 28 °C. (**A**) Pathogen-only, initial point; (**B**) Pathogen-only, 7 days; (**C**) Co-treatment with phage vB_PS-10, initial point; and (**D**) Co-treatment with phage vB_PS-10, 7 days.

## Data Availability

The data that support the findings of this study are available from the corresponding author upon reasonable request. The data are not publicly available due to privacy and intellectual property restrictions.

## Supplementary Materials

The following supporting information can be downloaded at: https://www.mdpi.com/article/10.3390/v18101061/s1, Figure S1: Plaque morphology of the bacteriophage isolated from agricultural samples in Phimai District, Nakhon Ratchasima Province, visualized on a *Pantoea* sp. isolate 2 bacterial lawn after 48 h of incubation at 28 °C. (A) Dried rice leaf; (B) Dired rice stubble; (C) Soil; (D) Dried rice roots; (E) Water 1; (F) Water 2. Figure S2: Effect of various MOI of vB_PS-10 on the growth of *Pantoae* sp. Isolate 2.
