# Supplementary material for: Bacteriophages Targeting an Emerging Pantoea spp. for Rice Disease Biocontrol
Source: Viruses. 2026 Sep 24;18(10):1061. doi: 10.3390/v18101061 (PMC13709549; doi:10.3390/v18101061)
Supplement: Supplementary file 1 [file viruses-18-01061-s001.zip › viruses-4518764-supplementary.pdf]

Supplementary Documents

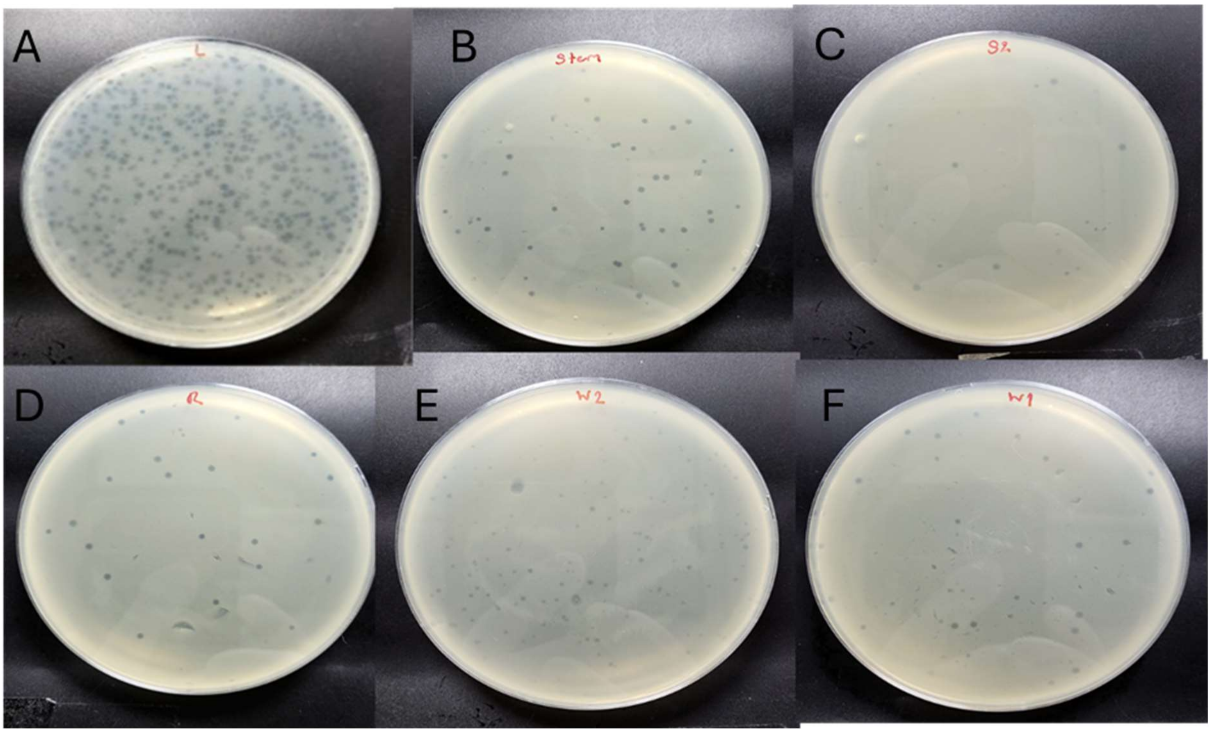

Figure S1. Plaque morphology of the bacteriophage isolated from agricultural samples in Phimai District, Nakhon Ratchasima Province, visualized on a *Pantoea* sp. isolate 2 bacterial lawn after 48 h of incubation at 28 °C. (A) Dried rice leaf; (B) Dired rice stubble; (C) Soil; (D) Dried rice roots; (E) Water 1; (F) Water 2.

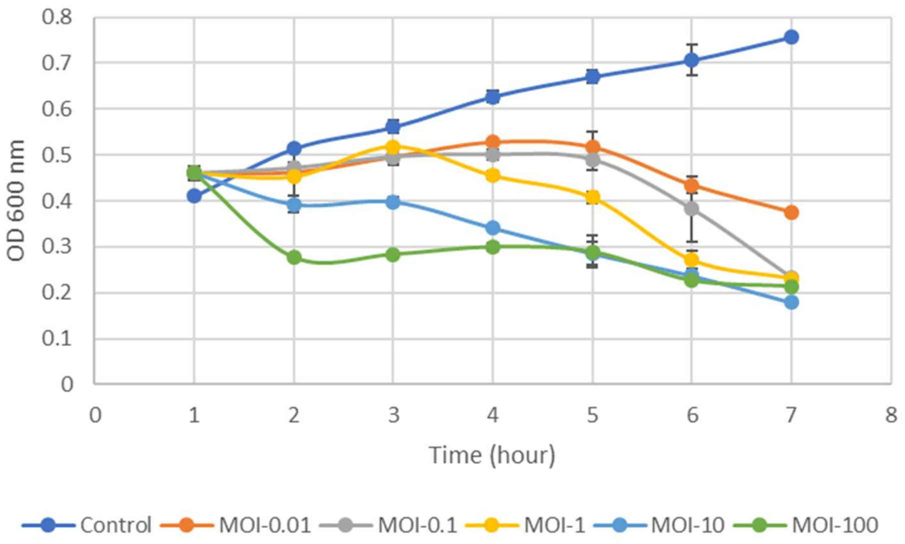

Figure S2. Effect of various MOI of vB_PS-10 on the growth of *Pantoae* sp. Isolate 2.
